# Behavioral Deficits in Juvenile Onset Huntington’s Disease

**DOI:** 10.3390/brainsci10080543

**Published:** 2020-08-11

**Authors:** Kathleen E. Langbehn, Ashley M. Cochran, Ellen van der Plas, Amy L. Conrad, Eric Epping, Erin Martin, Patricia Espe-Pfeifer, Peg Nopoulos

**Affiliations:** 1 Department of Psychiatry, University of Iowa Carver College of Medicine, Iowa City, IA 52242, USA; ashley-cochran@uiowa.edu (A.M.C.); ellen-vanderplas@uiowa.edu (E.v.d.P.); eric-epping@uiowa.edu (E.E.); erin-martin@uiowa.edu (E.M.); patricia-espepfeifer@uiowa.edu (P.E.-P.); 2 Stead Family Department of Pediatrics, University of Iowa Carver College of Medicine, Iowa City, IA 52242, USA; amy-l-conrad@uiowa.edu; 3 Department of Neurology, University of Iowa Carver College of Medicine, Iowa City, IA 52242, USA

**Keywords:** Huntington’s disease, behavioral regulation, executive function, trinucleotide repeat disorder

## Abstract

Reports of behavioral disturbance in Juvenile-Onset Huntington’s Disease (JOHD) have been based primarily on qualitative caregiver reports or retrospective medical record reviews. This study aims to quantify differences in behavior in patients with JOHD using informant- and self-report questionnaires. Informants of 21 children/young adults (12 female) with JOHD and 115 children/young adults (64 female) with a family history of Huntington’s Disease, but who did not inherit the disease themselves (Gene-Non-Expanded; GNE) completed the Behavior Rating Inventory of Executive Function (BRIEF) and the Pediatric Behavior Scale (PBS). Mixed linear regression models (age/sex adjusted) were conducted to assess group differences on these measures. The JOHD group had significantly higher scores, indicating more problems, than the GNE group on all BRIEF subscales, and measures of Aggression/Opposition and Hyperactivity/Inattention of the PBS (all *p* < 0.05). There were no group differences in Depression/Anxiety. Inhibit, Plan/Organize, Initiate, and Aggression/Opposition had significant negative correlations with Cytosine-Adenine-Guanine (CAG) repeat length (all *p* < 0.05) meaning that individuals with higher CAG repeats scored lower on these measures. There was greater discrepancy between higher informant-vs. lower self-reported scores in the JOHD group, supporting the notion of lack of insight for the JOHD-affected group. These results provide quantitative evidence of behavioral characteristics of JOHD.

## 1. Introduction

Huntington’s disease (HD) is an autosomal dominant neurodegenerative disease caused by a Cytosine-Adenine-Guanine (CAG) repeat expansion of the *Huntingtin* gene (*HTT*). The disease typically presents in adulthood with an average age of onset of 40 (referred to as Adult Onset HD or AOHD), and is marked by a combination of motor, cognitive, and behavioral symptoms. Approximately 1–10% of patients with HD will experience onset of symptoms before age 21, which is categorized as Juvenile-Onset HD (JOHD) [1].

Evidence of behavioral disturbances in JOHD is primarily predicated on retrospective medical record analyses [2,3] and caregiver reports [4,5,6] with few attempts to systematically evaluate these changes prospectively. Behavioral problems and cognitive decline are often among the first symptoms to present in individuals with JOHD [6,7,8,9] and can emerge years before the onset of motor symptoms [5], a pattern that is parallel with that of AOHD. Behavioral issues reported in JOHD include violence, aggression, oppositional behavior, obsession, depression, anxiety, impulsivity, attention issues, psychosis, and substance abuse [1,2,3,4,8,10,11]. Family members and caregivers of individuals with JOHD have reported that behavioral symptoms are often more distressing and disruptive than motor symptoms [6].

The Kids-HD and Kids-JOHD study are parallel programs at the University of Iowa. The Kids-HD study enrolls children/young adults (ages 5–26 years old) who are at-risk for HD based on a parent or grandparent having been diagnosed with HD. These children are genotyped for research purposes only, and categorized into the Gene-Expanded (GE, CAG > 36) or Gene Non-Expanded (GNE, CAG < 35) group. Those that are GE will go on to develop AOHD later in life and those that are GNE will never develop HD. The Kids-JOHD study enrolls children/young adults (ages 5–26 years old) who are already symptomatic with JOHD and have had molecular confirmation of the gene expansion (motor diagnosis made prior to age 21). The GNE group makes an excellent comparison group for the JOHD sample given that although they did not inherit the gene, they are from a family in which a parent, and possibly other family members, are suffering from HD, a family environment similar to the JOHD participants. The first aim of the current study was to establish group differences between JOHD and GNE participants based on informant ratings of behavior. The second aim was to examine a potential CAG repeat length effect in the JOHD participants. The third aim was to explore differences between self-reported and informant-reported executive function in participants over the age of 18.

## 2. Materials and Methods

### 2.1. Participants

This sample consists of children and young adults (ages 5–26) who participated in the Kids-HD or Kids-JOHD studies at the University of Iowa. Details of the Kids-HD program can be found in the 2019 publication on brain development in the Kids-HD sample [12]. From the children/young adults at risk from the Kids-HD study, we utilized the Gene-Non-Expanded (GNE) as controls.

Recruitment for the Kids-JOHD study was done through the Center of Excellence at the University of Iowa and national Huntington’s Disease Society of America events. To be eligible, participants required a genetic confirmation of an expanded CAG repeat. If the participant was older than 21 at the time of assessment, they were required to have had a clinical diagnosis prior to the age of 21.

All participants were recruited from across the United States and travelled to the University of Iowa to complete the study. The study followed an accelerated longitudinal design (ALD), where some individuals were assessed once, while others were assessed on multiple occasions with variable lengths of follow-up [12]. This design is less affected by attrition, which is especially important in this population due to disease progression. It also allows us to study the disease over a larger age range, and thus a greater number of participants. However, each age range may not be equally represented [13]. Participants completed multiple visits if they were willing and able to return for follow-up.

Both study protocols were approved by the Institutional Review Board (IRB) at the University of Iowa and were conducted in accordance with the Declaration of Helsinki. Parents or legally authorized representatives provided written consent for participants under the age of 18 or those who were unable to provide consent due to disease progression. Participants 18 years of age or older with the capacity to consent provided written informed consent for participation. This project was initially approved by the University of Iowa Institutional Review Board (IRB Number: 201109879) on 9 January 2012, and most recently approved on 15 May 2020.

### 2.2. Genetic Analysis

A DNA sample of either blood or saliva was obtained from participants in the Kids-HD study. The presence or absence of the mutant CAG expansion was determined using PCR analysis by the University of Iowa Molecular Diagnostic Laboratory. This analysis was done for research purposes only, and results were not disclosed to anyone including the participants, participants’ families, or the clinical research study staff [12]. All the participants in the Kids-JOHD study had to have molecular confirmation prior to enrollment and came with medical record documentation of the gene expansion.

### 2.3. Motor Rating

Like AOHD, the diagnosis of JOHD requires the presence of significant motor abnormality. A few of the participants in the JOHD study had been tested locally, yet examination by the neurologist locally showed no significant, or only subtle, motor findings. Our aim was to examine a homogenous group of motor-manifest patients. To that end, all participants were assessed by a trained motor examiner using the Unified Huntington’s Disease Rating Scale (UHDRS). A total motor score (TMS) was obtained by summing the core UHDRS items. To be included in the analysis, the JOHD participants were required to have a TMS of greater than 18. The rationale for the relatively high cut-off for the TMS is because the UHDRS is sensitive to developmental motor changes such that normal developing younger children will show higher scores than older children. Therefore, even in a large cohort of children at risk, but who did not inherit the gene expansion (Gene Non-Expanded or GNE), the UHDRS can be as high as 17, as shown by our previous analysis of the Kids-HD cohort [14]. In the current cohort, the highest TMS was 17 for a GNE participant. This was used as a guide for the JOHD group where the cut-off was determined to be 18 (with lowest TMS in the JOHD group being 19).

### 2.4. Behavioral Measures

Behavior Rating Inventory of Executive Function (BRIEF). While some standardized tests are designed to measure specific executive functioning skills in an individual, the BRIEF is a questionnaire designed to assess executive function behaviors [14]. Items are coded as “never”, “sometimes”, or “often” being a problem. The total Global Executive Composite score is divided into the Behavioral Regulation Index (BRI; Inhibit, Shift, Emotional Control) and the Metacognition Index (MI; Initiate, Working Memory, Plan/Organize, Organization of Materials, Monitor). Higher scores indicate more problematic behaviors.

If the participant was younger than 18 years old, the informant completed the BRIEF-Parent form; however, if the participant was 18 years old or older, the informant completed the BRIEF-Adult (BRIEF-A) Informant form. Informants were individuals who accompanied the participant to the research appointment. For participants younger than 18 years of age, the informant was a parent or legal guardian. For participants 18 years of age or older, the informant was a parent, guardian, spouse/partner, or friend. Additionally, participants age 18 and older completed the BRIEF-A Self Report Form. 

With regard to the Pediatric Behavior Scale-30 (PBS-30), the PBS is a parent/informant-report measure designed to assess broad domains of functioning; it was derived from the full 165-item version of the PBS [15]. The PBS-30 includes 30 Likert-scale items (“almost never or not at all” to “very often or very much”) that describe different behaviors, where higher scores reflect more problems. Four scales are calculated: Aggression/Opposition, Hyperactivity/Inattention, Depression/Anxiety, and Physical Health. The same form was used for all ages in the current study.

Supplementary Tables S1 and S2 summarize the number of observations available for each BRIEF and PBS subscale. Supplementary Table S3 summarizes the differences in scores between informant and self-reports for the BRIEF-A.

### 2.5. Statistical Analyses

Raw scores from the two scales were analyzed across the two groups via mixed linear regression models with individual BRIEF and PBS subscales as the outcome variables. Group, age, and sex were included as main effects in all regression models, and participant ID and family ID were included as random effects to account for non-independency of the observations. Including family ID in the model controlled for the effects of having more than one person from a family. Adding the participant ID controls meant that the correlation between repeat visits from a single individual was controlled for in the model. Sex by group interactions were entered into the model and subsequently removed if not significant.

The impact of CAG repeat length was examined in the JOHD group for measures that were significantly different between groups using mixed linear regression models to predict behavioral and executive functioning outcomes by CAG repeat expansion length.

Differences in BRIEF-A informant and self-reported scores were analyzed in patients 18 and older by calculating the difference in informant-reported and self-reported raw scores for each BRIEF-A subscale outcome measure. Difference scores were predicted with mixed linear effects models including main effects of group, age, and sex and controlling random effects of family ID. The difference score represents a difference in perspective on behavioral/cognitive problems between the participant and their parent. Thus, difference scores close to 0 represent agreement between self and proxy assessments, while large differences represent incongruent perspectives. All models were corrected for multiple comparisons using the False Discovery Rate (FDR) method. All analyses were completed using RStudio version 1.2.5042 (RStudio, PBC, Boston, MA, USA).

## 3. Results

### 3.1. Sample

The sample included 21 JOHD individuals (12 female). From this group, nine participants were seen once, nine were seen twice, two were seen three times, one was seen four times, and three were examined on five occasions, for a total of 49 observations. 

There were 115 GNE individuals (64 female). From this group, 60 participants were seen once, 30 were seen twice, 17 were seen three times, and 8 were seen for 4 visits for a total of 203 observations. There were no significant differences in distribution of sex between JOHD and GNE individuals (χ^2^ (1, *N* = 137) = 1.10 × 10^−31^, *p* = > 0.99). Average elapsed time between follow-up visits was 1.3 years (SD = 1.8 years). All participants were seen between March 2006 and February 2020 with an average of 1.65 visits and median of one visit.

Mean age at evaluation was significantly different between groups: JOHD patients were 15.23 years old on average (SD = 5.55) and GNE individuals were 13.47 years old on average (SD = 3.87 years; t (126.66) = 2.03), *p* = 0.04). CAG repeats ranged from 15 to 34 in the GNE group (median = 19) and from 54 to 102 in the JOHD group (median = 76). Distribution of total motor impairment scores (sum of core UHDRS items) ranged from 19 to 103 in the JOHD group. Average disease duration (defined as age at time of assessment minus age at time of clinical diagnosis) for individuals with JOHD was 3.6 years (SD = 1.5 years), meaning that most JOHD participants were early in the course of the motor manifest stage of the disease. Full group statistics are shown in Table 1.

### 3.2. Behavioral Performance Group Differences

**Figure 1:**
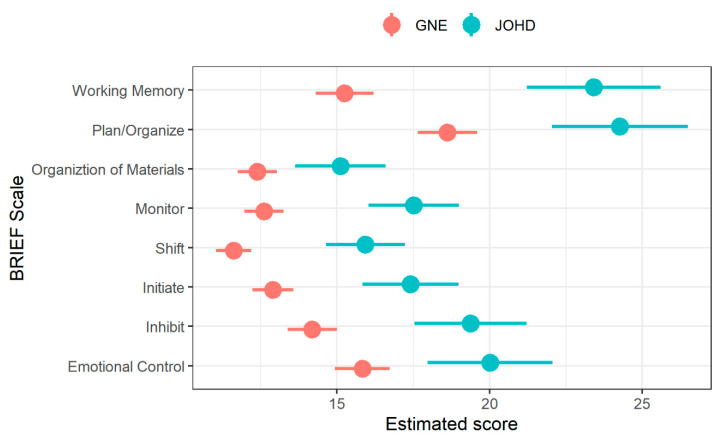
Differences in Behavior Rating Inventory of Executive Function (BRIEF) subscale scores between JOHD (blue) and GNE (red) participants. The x-axis shows age- and sex-adjusted estimates from mixed linear effects models after controlling for random effects of repeated measures and family ID. The y-axis shows subscales of the Behavioral Rating Inventory of Executive Function (BRIEF). The larger circles represent the means and horizontal lines indicate 95% confidence limits.

The JOHD group had statistically significantly higher scores than the GNE group on all subscales of the BRIEF (Emotional Control, Inhibit, Shift, Monitor, Plan/Organize, and Working Memory all FDR < 0.001; Organization of Materials FDR = 0.0015; see Figure 1 and Table 2). 

**Figure 2:**
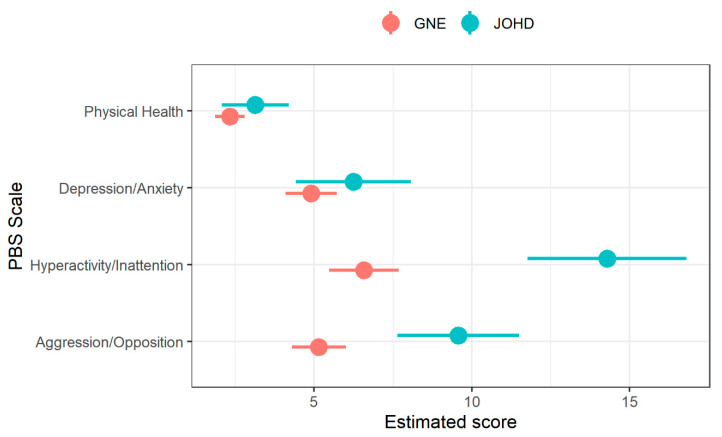
Differences in Pediatric Behavior Scale (PBS) subscale scores between JOHD (blue) and GNE (red) participants. The x-axis shows age- and sex-adjusted estimates from mixed linear effects models after controlling for random effects of repeated measures and family ID. The y-axis shows subscales of the Pediatric Behavior Scale-short form (PBS). The larger circles represent the means and horizontal lines indicate 95% confidence limits.

The JOHD group had statistically significantly higher scores than the GNE group on the Aggression/Opposition and Hyperactivity/Inattention subscales of the PBS (FDR = 0.00017 and <0.0001 respectively; see Figure 2 and Table 2). In contrast, there were no significant group differences in parent reported measures of Depression/Anxiety and Physical Health subscales of the PBS (both FDR > 0.1). There was no significant main effect of sex for any measure.

### 3.3. Genetic Expansion Correlations

Within JOHD, all measures had a negative correlation with CAG repeat length, however this reached significance for BRIEF Inhibit (*p* = 0.048), Plan/Organize (*p* = 0.034), and Initiate (*p* = 0.013) subscales of the BRIEF and the Aggression/Opposition (*p* = 0.038) scale of the PBS (see Table 3). A negative association indicates that JOHD participants with the highest CAG repeat tended to have the lowest behavioral scores. All other BRIEF and PBS subscales were not significantly predicted by CAG repeat length.

### 3.4. BRIEF-A Report Type Differences

**Figure 3:**
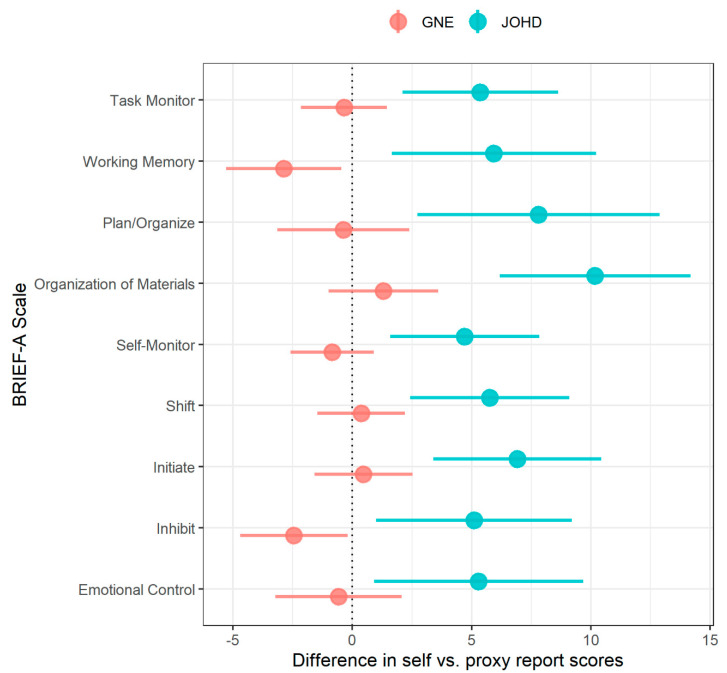
Differences in discrepancies between informant- and self-reported BRIEF-Adult (BRIEF-A) subscale scores between JOHD (blue) and GNE (red) participants. The x-axis shows the difference between informant and self-reported scores (calculated as informant—self) on the Behavioral Rating Inventory of Executive Function-Adult form (BRIEF-A); age- and sex-adjusted estimates are plotted from mixed linear effects models after controlling for random effects of family ID. The larger circles represent the means and horizontal lines indicate 95% confidence limits. The vertical black line at 0 marks no difference between informant and self-report scores.

In total, there were 35 observations for BRIEF-A (JOHD = 13, GNE = 22; see Supplementary Table S3). The difference score in the GNE group was generally close to 0, except for Inhibit (*p* = 0.003) and Working Memory (*p* = 0.002), where informants reported more problems than participants. In contrast, in the JOHD group the difference scores were consistently different from 0, with informants reporting more problems than JOHD patients (all FDR < 0.05; see Figure 3 and Table 4). Age and sex did not have significant effects on the difference between informant- and self-reported scores.

## 4. Discussion

The Kids-JOHD study is the first ever prospective, longitudinal study of this ultra-rare population. Therefore, this is the first analysis of behavioral symptoms of JOHD that measures behavior on a continuum, rather than using reports of behavioral issues from retrospective analyses of medical records or qualitatively by parents and caretakers of these patients [3,5,8,9,11,16]. Our findings provide quantitative support for the notion that behavioral dysfunction is prevalent among persons with JOHD [3,5,8,9,11,16]. Specific commonly reported symptoms in JOHD include aggressive and oppositional behavior, and difficulties with attention; consistent with this, the largest group differences we found were in parental reports of Hyperactivity/Inattention followed by Aggression/ Opposition [3,5,8,10,16]. 

Importantly, JOHD patients did not exhibit significant anxiety and depression. Different from externalizing behaviors that are easy for others to see, such as aggression and impulsivity, internalizing behaviors associated with subjective feelings of mood and being nervous may be harder to rate objectively [17]. Regardless of the inherent issues in parent reports of internalizing symptoms, it is clear that these symptoms were no more frequent in the JOHD subjects compared to the GNE children, supporting the notion that internalizing behaviors are not significantly affected in JOHD.

In the present study, mutant *HTT* CAG repeat expansion length was negatively correlated with all measures, but reached statistical significance with Inhibit, Plan/Organize, Initiate, and Aggression/Opposition indicating that patients with longer repeat lengths (typically resulting in childhood-onset JOHD [18]) exhibit fewer problems in these domains, while patients with short repeats (typically resulting in adolescent-onset JOHD [18]) exhibit more problem behaviors. These findings align with current reports that older-onset JOHD patients exhibit more behavioral issues than younger-onset JOHD patients [7]. In addition, opposition and aggression behavior normally peak in adolescence; therefore, an active brain disease during a time in which these behaviors normally peak may be one potential rationale for the adolescent onset having greater behavioral disturbance. However, in review of the reports from caregivers of those with childhood onset, problems with aggression and opposition were common early in the course of the disease, years prior to diagnosis. It may be that by the time of motor onset, the childhood onset patients have moved past a period of externalizing behavior [1,7].

Analyses evaluating differences between informant and self-reported measures of behavioral regulation and executive function indicated a possible lack of insight among JOHD patients in their behaviors. While the GNE group had similar scores between informant and self-reports, the JOHD participants consistently rated themselves as having fewer behavioral and executive functioning problems than what was reported by their informants. Limited insight into behavioral and cognitive difficulties is a known feature in patients with AOHD [19]. These results suggest that informant ratings are crucial when quantifying behavioral and executive dysfunction in JOHD.

Parents often report that cognitive and behavior issues are the first harbinger of change and can occur sometimes years prior to final motor diagnosis. Some families in the JOHD community have lobbied for using behavioral changes as a diagnostic criteria for disease [8]. This would be inappropriate for several reasons. Any symptom utilized for diagnosis has to be sensitive and specific to the disease. Although all of the subjects here are already motor manifest, it is important to point out that behavioral symptoms are not present in all patients, therefore these behavioral ratings are not sensitive to the presence of JOHD. Secondly elevated behavioral ratings are not specific to JOHD [6]. There were many children in the GNE group with elevated scores (in fact the two highest scores in the entire sample on hyperactivity and inattention from the PBS came from GNE participants). Although as a group, the JOHD sample had elevated scores compared to GNE, the presence of elevated scores in any one individual is not specific to JOHD. This underscores the notion that changes in behavior should not be utilized for diagnostic purposes for children at risk for AOHD or JOHD.

This study was not without limitations. First, with JOHD being an extremely rare disorder affecting only 1–10% of individuals with HD [1], our sample was limited to 21 individuals with JOHD; however, we leveraged an accelerated longitudinal design to increase the number of observations. Second, parent and informant ratings are objective measures of behavior. Since parents of JOHD are aware of their diagnosis, they may be biased in reporting their child as having greater symptoms, simply knowing that it is commonly known amongst these families that behavioral disturbances occur in children with JOHD. Finally, since self-reported measures were not used in this study for individuals younger than 18, we relied on a small sample for our self vs. informant analyses.

## 5. Conclusions

In this study, patients with juvenile-onset Huntington’s Disease (JOHD) exhibited significant behavioral problems relative to gene non-expanded (GNE) counterparts. Those participants with longer CAG repeats (earlier age of onset) had fewer behavioral problems compared to those with relatively shorter repeats (later age of onset). Lack of insight may have prohibited adult patients with JOHD from providing reliable assessments of their behavioral problems. Further research should be conducted with larger samples to create a JOHD-specific behavioral rating measure including both self and proxy measures that may be used as markers for clinical trials and treatments.

## Figures and Tables

**Table 1:** Demographics by groups.

	GNE (*N* = 203)	JOHD (*N* = 49)
Age		
Mean (SD)	13.5 (3.87)	15.2 (5.55)
Median (Min, Max)	13.7 (6.00, 22.5)	15.8 (5.08, 25.1)
Sex		
Females	120 (59.1%)	29 (59.2%)
Males	83 (40.9%)	20 (40.8%)
CAG		
Median (Min, Max)	19.0 (15.0, 34.0)	76.0 (54.0, 102)
TMS		
Mean (SD)	1.28 (2.67)	58.0 (21.3)
Median (Min, Max)	0 (0, 17.0)	55.0 (19.0, 103)

Note: GNE, Gene-Non-Expanded, i.e., participants with a family history of Huntington’s Disease who did not inherit the mutant expansion; JOHD, participants with juvenile onset Huntington’s Disease; CAG, CAG repeat expansion length; TMS, total motor score, calculated as cumulative Unified Huntington’s Disease Rating (UHDRS) items.

**Table 2:** BRIEF and PBS model statistics and marginal means.

Variable	Diff Means	*t*-Value (df)	FDR	Marg Mean GNE	95% CI GNE	Marg Mean JOHD	95% CI JOHD
Emotional Control	−4.19	*t*_(120)_ = −3.68	0.000466 *	15.83	14.93: 16.73	20.02	17.96: 22.08
Inhibit	−5.18	*t*_(120)_ = −5.1	2.62 × 10^−6^ *	14.19	13.39: 15.00	19.37	17.53: 21.21
Initiate	−4.5	*t*_(117)_ = −5.16	2.43 × 10^−6^ *	12.91	12.23: 13.58	17.41	15.82: 19.00
Shift	−4.31	*t*_(110)_ = −5.99	1.19 × 10^−7^ *	11.62	11.04: 12.19	15.93	14.63: 17.23
Self-Monitor	−4.89	*t*_(112)_ = −5.96	1.19 × 10^−7^ *	12.62	11.98: 13.26	17.51	16.02: 19.00
Organization of Materials	−2.72	*t*_(115)_ = −3.32	0.00147 *	12.40	11.75: 13.04	15.12	13.63: 16.60
Plan/Organize	−5.65	*t*_(115)_ = −4.58	2.02 × 10^−5^ *	18.62	17.64: 19.60	24.27	22.04: 26.50
Working Memory	−8.16	*t*_(120)_ = −6.73	7.42 × 10^−9^ *	15.25	14.30: 16.21	23.41	21.21: 25.61
Aggression/Opposition	−4.42	*t*_(119)_ = −4.11	0.000109 *	5.15	4.30: 6.01	9.57	7.63: 11.51
Hyperactivity/Inattention	−7.7	*t*_(117)_ = −5.51	6.5 × 10^−7^ *	6.58	5.47: 7.69	14.28	11.75: 16.80
Depression/Anxiety	−1.34	*t*_(95.8)_ = −1.31	0.192	4.91	4.09: 5.73	6.25	4.41: 8.08
Physical Health	−0.798	*t*_(102)_ = −1.35	0.192	2.34	1.87: 2.81	3.14	2.07: 4.21

Note: BRIEF and PBS raw scores are reported. Diff Means indicates the difference in group mean estimates. Marg Mean indicates the estimated marginal means for each group. Abbreviations: GNE, Gene-Non-Expanded group; JOHD, Juvenile-Onset Huntington’s Disease group. * indicates False Discovery Rate (FDR) *p*-adjusted < 0.0005.

**Table 3:** Genetic expansion effects in JOHD.

Variable	Coefficient	95% CI	*t*-Value (df)	*p*-Value
Emotional Control	−0.08	−0.35: 0.19	*t*_(47)_ = −0.615	0.548
Inhibit	−0.23	−0.45: −0.01	*t*_(47)_ = −2.08	0.0479 *
Shift	−0.04	−0.18: 0.11	*t*_(47)_ = −0.493	0.627
Working Memory	−0.24	−0.53: 0.04	*t*_(47)_ = −1.71	0.102
Plan/Organize	−0.26	−0.50: −0.03	*t*_(47)_ = −2.24	0.0343 *
Initiate	−0.18	−0.32: −0.05	*t*_(46)_ = −2.68	0.0126 *
Self-Monitor	−0.16	−0.33: 0.01	*t*_(46)_ = −1.87	0.0723
Organization of Materials	−0.15	−0.36: 0.06	*t*_(46)_ = −1.43	0.166
Aggression/Opposition	−0.30	−0.57: −0.03	*t*_(47)_ = −2.21	0.0379 *
Hyperactivity/Inattention	−0.23	−0.51: 0.05	*t*_(47)_ = −1.68	0.114

Note: Statistics based on measures that were significantly different between GNE and JOHD groups. JOHD, Juvenile-Onset Huntington’s Disease group. Higher means indicate more behavioral/executive problems on the BRIEF and PBS. * indicates *p*-value < 0.05.

**Table 4:** BRIEF-A model statistics and marginal means.

Variable	Diff Means	*t*-Value (df)	FDR	Marg Mean GNE	95% CI GNE	Marg Mean JOHD	95% CI JOHD
Emotional Control	−5.88	*t*_(18.6)_ = −2.13	0.0466 *	−0.58	3.25: 2.09	5.30	0.73: 9.87
Inhibit	−7.55	*t*_(25.1)_ = −3.06	0.0117 *	−2.45	−4.72: −0.18	5.10	0.96: 9.24
Initiate	−6.45	*t*_(20.4)_ = −2.96	0.0117 *	0.47	−1.60: 2.54	6.92	3.29: 10.54
Shift	−5.39	*t*_(25.2)_ = −2.68	0.0145 *	0.37	−1.48: 2.22	5.76	2.38: 9.13
Self-Monitor	−5.56	*t*_(24.6)_ = −2.93	0.0117 *	−0.85	−2.60: 0.90	4.71	1.55: 7.88
Organization of Materials	−8.88	*t*_(20.7)_ = −3.6	0.0113 *	1.29	−1.02: 3.61	10.18	6.07: 14.29
Plan/ Organize	−8.18	*t*_(25.5)_ = −2.71	0.0145 *	−0.38	−3.17: 2.41	7.80	2.67: 12.93
Working Memory	−8.81	*t*_(24.4)_ = −3.37	0.0113 *	−2.87	−5.30: −0.45	5.94	1.59: 10.28
Task Monitor	−5.71	*t*_(24.9)_ = −2.89	0.0117 *	−0.35	−2.16: 1.47	5.37	2.06: 8.67

Note: Statistics based on group differences between the difference in BRIEF-Adult report type, as calculated by informant-report scores—self-report scores. Diff Means indicates the difference in group mean estimates. Marg Mean indicates the estimated marginal means for each group. GNE, gene-non-expanded group; JOHD, juvenile-onset Huntington’s Disease group. Higher means indicate more behavioral/executive problems on the BRIEF-A. * indicates FDR *p*-adjusted < 0.05.

## Supplementary Materials

The following are available online at https://www.mdpi.com/2076-3425/10/8/543/s1, Table S1: BRIEF subscale statistics, Table S2: PBS subscale statistics, Table S3: BRIEF-Adult subscale statistics.
