# Supplementary material for: Behavioral Deficits in Juvenile Onset Huntington’s Disease
Source: Brain Sci. 2020 Aug 11;10(8):543. doi: 10.3390/brainsci10080543 (PMC7464355; doi:10.3390/brainsci10080543)
Supplement: Supplementary file 1 [file brainsci-10-00543-s001.zip › supplementary table 2- PBS subscales.docx]

Supplementary Table 2. PBS subscales

|  | **GNE (N=203)** | **JOHD (N=49)** |
| --- | --- | --- |
| **Aggression/Opposition** |  |  |
| Mean (SD) | 4.89 (4.36) | 8.18 (6.46) |
| Median [Min, Max] | 4.00 [0, 18.0] | 7.00 [0, 21.0] |
| **Hyperactivity/Inattention** |  |  |
| Mean (SD) | 6.02 (6.03) | 13.2 (7.13) |
| Median [Min, Max] | 4.00 [0, 27.0] | 13.0 [0, 25.0] |
| **Depression/Anxiety** |  |  |
| Mean (SD) | 4.72 (4.55) | 5.24 (4.78) |
| Median [Min, Max] | 3.00 [0, 21.0] | 4.00 [0, 21.0] |
| **Physical Health** |  |  |
| Mean (SD) | 2.12 (2.55) | 2.59 (2.81) |
| Median [Min, Max] | 1.00 [0, 15.0] | 2.00 [0, 14.0] |

PBS subscale statistics. Mean, standard deviation, median, and range calculated for each PBS subscale for both groups. Abbreviations: GNE, gene-non-expanded group; JOHD, Juvenile-Onset Huntington’s Disease group.
