# Supplementary material for: Behavioral Deficits in Juvenile Onset Huntington’s Disease
Source: Brain Sci. 2020 Aug 11;10(8):543. doi: 10.3390/brainsci10080543 (PMC7464355; doi:10.3390/brainsci10080543)
Supplement: Supplementary file 1 [file brainsci-10-00543-s001.zip › supplementary table 3- BRIEF-A score differences.docx]

Supplementary Table 3. Difference between informant and self BRIEF-A reports

|  | **GNE (N=24)** | **JOHD (N=13)** |
| --- | --- | --- |
| **Emotional Control** |  |  |
| Mean (SD) | -0.955 (4.91) | 5.58 (6.11) |
| Median [Min, Max] | -2.00 [-11.0, 11.0] | 7.50 [-7.00, 15.0] |
| **Inhibit** |  |  |
| Mean (SD) | -2.36 (3.32) | 5.08 (6.42) |
| Median [Min, Max] | -3.00 [-11.0, 6.00] | 6.00 [-6.00, 13.0] |
| **Initiate** |  |  |
| Mean (SD) | 0.0455 (4.02) | 7.67 (4.42) |
| Median [Min, Max] | 0 [-7.00, 8.00] | 8.00 [-2.00, 12.0] |
| **Shift** |  |  |
| Mean (SD) | 0.500 (3.14) | 5.42 (4.89) |
| Median [Min, Max] | 0 [-4.00, 7.00] | 6.50 [-5.00, 11.0] |
| **Self-Monitor** |  |  |
| Mean (SD) | -0.682 (2.78) | 4.58 (5.07) |
| Median [Min, Max] | -1.00 [-5.00, 7.00] | 4.50 [-5.00, 11.0] |
| **Organization of Materials** |  |  |
| Mean (SD) | 1.36 (4.89) | 10.0 (4.63) |
| Median [Min, Max] | 1.50 [-9.00, 12.0] | 9.50 [5.00, 16.0] |
| **Plan/Organize** |  |  |
| Mean (SD) | -0.773 (4.53) | 9.08 (7.90) |
| Median [Min, Max] | -0.500 [-10.0, 8.00] | 8.50 [-4.00, 19.0] |
| **Working Memory** |  |  |
| Mean (SD) | -2.59 (3.36) | 5.42 (7.25) |
| Median [Min, Max] | -3.50 [-7.00, 7.00] | 5.00 [-7.00, 15.0] |
| **Task Monitor** |  |  |
| Mean (SD) | -0.227 (3.19) | 5.50 (4.87) |
| Median [Min, Max] | 0 [-6.00, 7.00] | 5.50 [-1.00, 11.0] |

BRIEF-Adult subscale statistics. Mean, standard deviation, median, and range calculated for each group for the difference between BRIEF-A report type, as calculated by informant-report scores – self-report scores. Higher values indicate more behavioral/executive problems rated by informants than participants. Abbreviations: GNE, Gene-Non-Expanded group; JOHD, Juvenile-Onset Huntington’s Disease group.
