# Supplementary material for: Behavioral Deficits in Juvenile Onset Huntington’s Disease
Source: Brain Sci. 2020 Aug 11;10(8):543. doi: 10.3390/brainsci10080543 (PMC7464355; doi:10.3390/brainsci10080543)
Supplement: Supplementary file 1 [file brainsci-10-00543-s001.zip › supplementary table 1- BRIEF subscales.docx]

Supplementary Table 1. BRIEF subscales

|  | **GNE (N=203)** | **JOHD (N=49)** |
| --- | --- | --- |
| **Emotional Control** |  |  |
| Mean (SD) | 15.6 (4.83) | 19.0 (6.30) |
| Median [Min, Max] | 15.0 [10.0, 30.0] | 19.0 [10.0, 30.0] |
| **Inhibit** |  |  |
| Mean (SD) | 14.0 (4.66) | 18.6 (5.69) |
| Median [Min, Max] | 13.0 [8.00, 30.0] | 20.0 [8.00, 30.0] |
| **Shift** |  |  |
| Mean (SD) | 11.5 (3.45) | 15.8 (3.47) |
| Median [Min, Max] | 11.0 [6.00, 23.0] | 15.0 [10.0, 24.0] |
| **Working Memory** |  |  |
| Mean (SD) | 15.1 (5.46) | 21.6 (5.96) |
| Median [Min, Max] | 13.0 [8.00, 30.0] | 21.0 [12.0, 49.0] |
| **Plan/Organize** |  |  |
| Mean (SD) | 18.4 (6.11) | 23.5 (5.73) |
| Median [Min, Max] | 17.0 [10.0, 36.0] | 24.0 [12.0, 36.0] |
| **Initiate** |  |  |
| Mean (SD) | 12.6 (3.84) | 17.1 (3.69) |
| Median [Min, Max] | 12.0 [8.00, 24.0] | 17.0 [10.0, 24.0] |
| **Monitor** |  |  |
| Mean (SD) | 12.6 (4.09) | 16.9 (4.31) |
| Median [Min, Max] | 12.0 [6.00, 24.0] | 17.0 [6.00, 24.0] |
| **Organization of Materials** |  |  |
| Mean (SD) | 12.3 (3.56) | 15.0 (5.20) |
| Median [Min, Max] | 12.0 [6.00, 22.0] | 14.5 [6.00, 24.0] |
| **Behavior Regulation Index** |  |  |
| Mean (SD) | 42.0 (11.5) | 56.8 (14.7) |
| Median [Min, Max] | 39.0 [28.0, 78.0] | 55.0 [33.0, 87.0] |
| **Metacognition Index** |  |  |
| Mean (SD) | 71.0 (20.1) | 92.5 (19.9) |
| Median [Min, Max] | 68.0 [40.0, 132] | 92.0 [38.0, 128] |
| **Global Executive Composite** |  |  |
| Mean (SD) | 113 (29.6) | 149 (30.8) |
| Median [Min, Max] | 106 [70.0, 210] | 150 [92.0, 210] |

BRIEF subscale statistics. Mean, standard deviation, median, and range calculated for each BRIEF subscale for both groups. Abbreviations: GNE, gene-non-expanded group; JOHD, juvenile-onset Huntington’s Disease group.
